# Incidence and Clinical Impact of Endocrinopathy Following First-Line Nivolumab-Plus-Relatlimab Therapy for Metastatic Melanoma

**DOI:** 10.3390/cancers18142349

**Published:** 2026-07-21

**Authors:** Julia Reitkopp, Wolfram Samlowski, Mahir Hasan

**Affiliations:** 1 Touro University Nevada College of Osteopathic Medicine, Henderson, NV 89104, USA; jreitkop@student.touro.edu (J.R.); mhasan11@student.touro.edu (M.H.); 2 Nevada Oncology Specialists, Las Vegas, NV 89148, USA; 3 Department of Internal Medicine, University of Nevada, Las Vegas (UNLV), Las Vegas, NV 89102, USA

**Keywords:** checkpoint inhibitor therapy, hypothyroidism, hypopituitarism, LAG-3 antibody, ipilimumab, nivolumab, PD-1 antibody

## Abstract

Background: Dual immune checkpoint inhibitor therapy with nivolumab plus relatlimab has substantial clinical activity against metastatic melanoma. The incidence and timing of endocrine immune-related adverse events with this treatment remain poorly characterized. Methods: We conducted a retrospective record review of 52 sequential patients who received nivolumab plus relatlimab as initial therapy for metastatic cutaneous melanoma. All patients underwent sequential endocrine screening (TSH, FT4, ACTH, cortisol) prior to each monthly treatment cycle. The incidence and onset of hypothyroidism and hypopituitarism were evaluated, as was cancer treatment outcome. Results: Biochemical evidence for endocrinopathy was identified in 25% of patients. This included a 13.5% incidence of hypothyroidism (median onset 79.0 ± 63.9 days) and 11.5% incidence of hypopituitarism (median onset 243.5 ± 75.6 days). Due to screening and early replacement therapy, there were no related hospitalizations. Patients who developed endocrinopathy showed a trend toward improved progression-free and overall survival. An exploratory analysis suggested that the incidence of endocrinopathy was significantly lower in patients treated with nivolumab plus relatlimab than in those treated with ipilimumab plus nivolumab. Conclusions: During treatment with nivolumab plus relatlimab, endocrinopathy developed in approximately 25% of metastatic melanoma patients, emphasizing a need for screening testing. In an exploratory analysis, endocrinopathy appeared less frequent than in ipilimumab-plus-nivolumab-treated patients. Recovery from endocrinopathy appeared uncommon. Development of delayed endocrinopathy following elective treatment discontinuation for patients in remission was rare (3.8%). Patients who developed endocrinopathy showed a trend toward improved clinical outcome.

## Simple Summary

Cancer immunotherapy using combinations of checkpoint inhibitor antibodies has shown high levels of clinical activity in the treatment of metastatic melanoma. These agents often produce complete remissions of wide-spread melanoma and long-term survival. All current cancer immunotherapy agents have the potential to trigger side effects related to excessive activation of the immune system. While many of these side effects are reversible, immune damage to endocrine organs may unfortunately be permanent. We evaluated how often the relatively new combination of nivolumab plus relatlimab caused problems with endocrine gland function. We found that low thyroid function occurred in about 13.5% of patients, with 11.5% developing low pituitary (“master gland”) function. These problems mostly occurred during the period of drug treatment but seemed to persist long-term. The frequency of these changes appeared to occur significantly less frequently than was observed following treatment with an alternative standard regimen consisting of ipilimumab plus nivolumab.

## 1. Introduction

Melanoma currently represents the fifth most common cancer among men and women. It is estimated that over 104,000 individuals will be diagnosed with invasive melanoma in 2025, resulting in 8430 deaths, due to the development of metastasis [1]. Prior to the development of modern therapy, the prognosis of metastatic melanoma was poor, with a median overall survival (OS) of 5.9 to 6.5 months [2,3,4,5]. The median progression-free survival rate (PFS) in phase II clinical trials from this era was only 1.7 months, with only 25% of patients alive at 1 year [4].

Significant progress in melanoma treatment occurred following the discovery of inhibitory immune checkpoints that regulate cytotoxic T cell function [6]. Monoclonal antibodies, termed immune checkpoint inhibitors (ICI), directed against these inhibitory receptors, activated immunologic recognition of cancers [7]. Combined blockade of CTLA-4 and PD-1, using ipilimumab plus nivolumab (I+N), resulted in substantial improvements in long-term PFS and melanoma specific survival [8]. At the 10-year follow-up, I+N produced a 31% PFS, which was superior to the PFS achievable with monotherapy using either nivolumab (23%) or ipilimumab (6%) [8]. Many of the clinical responses appeared quite durable, with a low risk of relapse after 3 years. Thus, dual checkpoint inhibitor therapy has become the standard initial treatment for most patients with metastatic melanoma.

More recently, a novel dual checkpoint inhibitor therapy targeting PD-1 plus another inhibitory immune checkpoint, LAG-3, has also demonstrated substantial clinical activity. Treatment with the monoclonal antibodies nivolumab and relatlimab (N+R) improved PFS and OS compared to nivolumab monotherapy [9]. This regimen has not been directly compared to I+N in a randomized phase III trial. However, in an ad hoc comparison, N+R seemed to have a similar level of effectiveness to the standard I+N regimen [10]. One potential advantage of the N+R combination appeared to be a substantial reduction in treatment-related toxicity. In this report 61% of patients treated with I+N developed grade 3 and 4 toxicities. In comparison, only 23% of patients treated N+R developed severe toxicities [10].

Endocrine organ damage is a relatively common checkpoint-inhibitor-associated immune-related adverse event (irAE). Most commonly, this manifests as primary hypothyroidism or hypopituitarism (secondary hypoadrenalism) [11,12]. More rarely, other endocrine glands (e.g., pancreas) can be affected. Like many other ICI-induced toxicities, endocrine toxicity usually seems to present relatively early during therapy. However, unlike most other acute toxicities, endocrinopathy seems to persist long-term following completion of treatment. Thus, the potential long-term need for endocrine replacement therapy needs to be considered in the risk-benefit assessment of ICI treatment.

Estimates of the incidence of endocrine toxicity with combination ICI therapy have been quite variable [11,12,13]. Patients in our clinic are routinely monitored on each visit for the development of endocrinopathy. We performed a retrospective analysis of patient data to more accurately define the incidence and timing of onset of biochemical evidence for hypothyroidism/hypopituitarism following N+R treatment. The effect of endocrinopathy on clinical outcome was monitored. We also performed an exploratory analysis comparing the incidence of endocrinopathy with N+R versus a similar-sized cohort of patients treated with I+N.

## 2. Materials and Methods

### 2.1. Patient Identification

In this retrospective data analysis, potential patients were identified by searching a Health Insurance Portability and Accountability Act (HIPAA)-compliant iKnowMed clinical database (IKM-G2, McKesson, Woodlands, TX, USA). This database was searched for patients who received N+R as initial treatment for metastatic cutaneous melanoma. All patients were treated by a single physician (W.S.). Patients with non-cutaneous melanoma (e.g., uveal or mucosal melanoma) were excluded from analysis, as were patients who never received any planned ICI treatment or only received a single dose. It should be noted than many adult patients have pre-existing hypothyroidism. We therefore included pre-existing endocrinopathy patients in our consecutive patient series to provide information on whether there was a potential for development of additional or worsening endocrine disorders during N+R treatment. Patients who had received prior PD-1 antibody treatment as adjuvant therapy were eligible for this analysis.

### 2.2. Data Extraction

Individual patient records were accessed, and relevant data was extracted from the medical record into a password-protected Excel spreadsheet (version 16.94, Microsoft Corporation, Redmond, WA, USA). To retain confidentiality, identifying information was deleted from the spreadsheet prior to analysis, and each subject was assigned a unique record number. Extracted demographic data included age, gender, date of birth (DOB), and race. In addition, potential driver mutation status and pretreatment lactate dehydrogenase (LDH) levels were also recorded. Patients who received prior adjuvant immunotherapy were noted, as was evidence for preexisting endocrinopathy. The date of onset of endocrinopathy was recorded, as was the timing and duration of immunotherapy. If the time of endocrinopathy diagnosis was after the end of immunotherapy treatment, patients were characterized as having a delayed onset. The overall survival rate and progression-free status were calculated from the initial ICI start date. Any additional ICI-induced toxicities were documented. This study design was reviewed by the Western (WGC) Institutional Review Board (IRB) chair and was deemed exempt from a full IRB review.

### 2.3. Treatment Regimens

All patients were treated with combination N+R immunotherapy. This treatment consisted of a fixed dose of nivolumab 480 mg plus relatlimab 160 mg intravenously over 30 min. Treatment was administered every 4 weeks. If immunologic toxicity was noted, N+R treatment was delayed until toxicity was controlled. There were no modifications of drug doses administered. Patients who achieved a confirmed complete remission underwent elective treatment discontinuation, based on an institutional protocol [14].

### 2.4. Identification of Endocrine Abnormalities

Medication records were also reviewed to identify the use of systemic corticosteroids or other agents that could potentially suppress the hypothalamic–pituitary–adrenal axis and confound the interpretation of cortisol measurements. Borderline cortisol or ACTH results were interpreted in relation to sequential laboratory testing and development of clinical symptoms (e.g., fatigue, decreased blood pressure, orthostatic symptoms) to indicate the need for endocrine replacement therapy. Routine endocrine monitoring in this retrospective analysis focused primarily on thyroid and adrenal axis testing (TSH, FT4, ACTH, and cortisol), while additional pituitary hormones (LH/FSH, prolactin, GH/IGF-1) were not systemically measured.

All patients had baseline endocrine testing prior to starting N+R therapy. Patients were routinely monitored for the development of endocrinopathy prior to each treatment (every 4 weeks). This included laboratory testing for thyroid stimulating hormone (TSH), free tetraiodothyronine (FT4), random adrenocorticotropic hormone (ACTH) and serum cortisol. The goal was not to quantitate diurnal variation in the hypothalamic-adrenal axis, but merely to screen for the presence of detectable hormone levels. Primary hypothyroidism was identified based on elevated TSH and low FT4. Transient elevations in FT4 and suppression of TSH were felt to indicate hyperthyroidism induced by autoimmune thyroiditis. Hypopituitarism was diagnosed based on low ACTH and cortisol levels, as well as low TSH and FT4. In contrast, no patients demonstrated evidence of primary adrenal insufficiency, which would be characterized by low cortisol with an elevated ACTH.

### 2.5. Response Assessment

Clinical responses in metastatic melanoma patients were evaluated as the best objective response rate (BORR) at 12 months [15], according to RECIST 1.1 criteria [16].

### 2.6. Statistical Assessment

Fundamental statistical analysis, including mean, median, and standard deviation, was calculated via an Excel spreadsheet. Overall survival (OS) and progression-free survival (PFS) rates were analyzed using the method of Kaplan and Meier [17]. Comparison of progression-free survival and overall survival were performed using Gehan–Breslow–Wilcoxon test [18,19]. Exploratory analysis of the percentage of patients developing endocrinopathy following I+N therapy versus N+R treatment were performed via Mann–Whitney test [20].

### 2.7. Artificial Intelligence Statement

No artificial intelligence tools were used in the development of this manuscript.

## 3. Results

### 3.1. Patient Demographics

We identified 52 sequential metastatic melanoma patients, treated in a consistent fashion with N+R by a single physician (WS) between June 2022 and February 2025. None of our patients were receiving glucocorticosteroids at baseline. The median age of patients at the start of treatment was 72.9 ± 14 years (range 39.2–92.6 years). There were 48 Caucasians (92.3%), two Hispanics (3.8%), one Asian (1.9%), and one Pacific Islander (1.9%). The median potential follow-up of patients in this series was 12.1 ± 8.6 months. Following treatment, 25 patients were alive with disease (48.1%), 18 patients had no evidence of disease (34.6%), and nine patients had died of metastatic melanoma (17.3%) during the follow-up interval.

### 3.2. Development of Endocrinopathy

**Figure 1:**
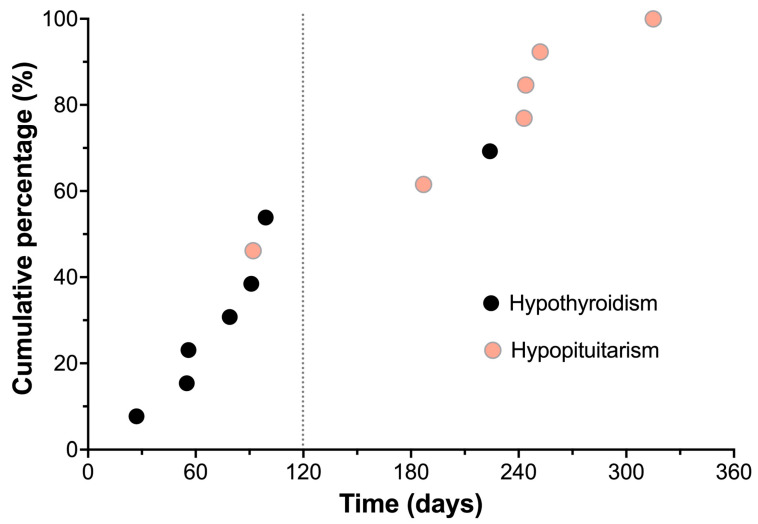
Time to onset (days) from start of nivolumab plus relatlimab therapy to development of hypothyroidism or hypopituitarism.

Thirteen patients (25%) developed either hypothyroidism or hypopituitarism based on serial laboratory evaluations. Most patients developed endocrinopathy within the first 9 months of N+R treatment. Individual patient clinical characteristics are described (Supplemental data Table S1). Seven patients (13.5%) developed primary hypothyroidism. Six additional patients (11.5%) developed hypopituitarism, associated with central hypothyroidism and secondary adrenal insufficiency. The median time to onset of primary hypothyroidism was 79.0 ± 63.9 days from the start of treatment. The onset of hypopituitarism during treatment was more delayed, at a median of 243.5 ± 75.6 days (Figure 1). None of our patients developed type 2 diabetes or primary adrenal insufficiency. Only two patients (3.85%) developed delayed onset of endocrinopathy following elective treatment discontinuation, while in remission, with a median onset of 127.25 ± 78.06 days. Treatment of endocrinopathy did not result in treatment delays or discontinuation.

It should be noted that baseline hypothyroidism was relatively frequent in our treatment population. Nine of 52 patients (17.3%) in our series had pre-existing hypothyroidism at the start of N+R therapy. We reviewed whether prior adjuvant immunotherapy may have contributed to this finding. However, only three of the nine patients with pre-existing hypothyroidism had received prior adjuvant immunotherapy. None of these three patients developed worsening endocrinopathy after treatment.

### 3.3. Effect of Endocrinopathy on Treatment Outcome

**Figure 2:**
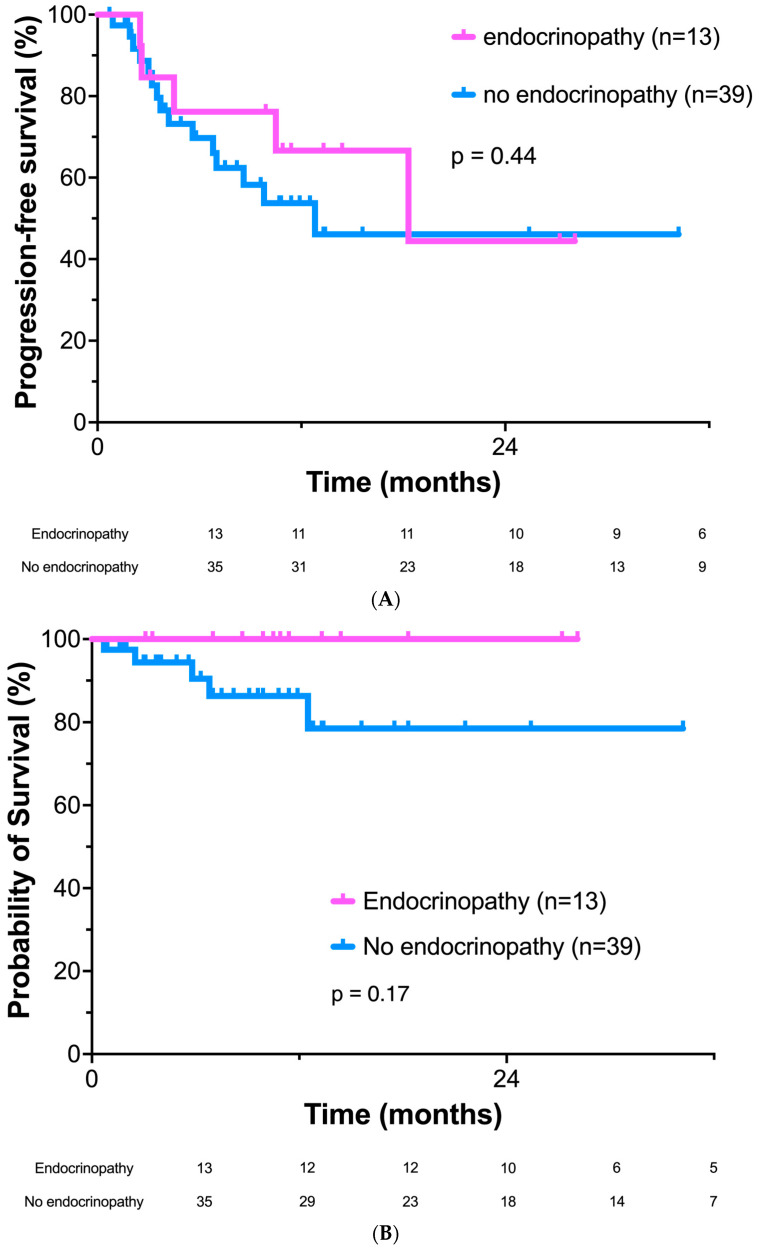
Clinical outcome of patients who developed endocrinopathy versus those who did not. The number of patients available for assessment at various times is indicated in the table below the figure. (**A**): progression-free survival; (**B**): overall survival. Hashmarks indicate censored patients.

There is a suspicion that the development of immune side-effects from ICI treatment may improve anticancer responses. We therefore evaluated whether the development of endocrinopathy following N+R therapy affected progression-free and overall survival (Supplemental data Table S1). Patients who developed endocrinopathy trended toward improved PFS compared to patients who did not develop endocrinopathy (Figure 2A). Similarly, patients who developed endocrinopathy showed a trend toward improved overall survival (Figure 2B). Neither trend reached statistical significance (*p* = 0.44 and 0.17, respectively). Overall, approximately 50% of N+R treated patients achieved a confirmed complete remission and were able to undergo elective treatment discontinuation, based on an institutional protocol [14].

### 3.4. Exploratory Analysis of the Incidence of Endocrinopathy Following N+R vs. I+N Treatment

**Figure 3:**
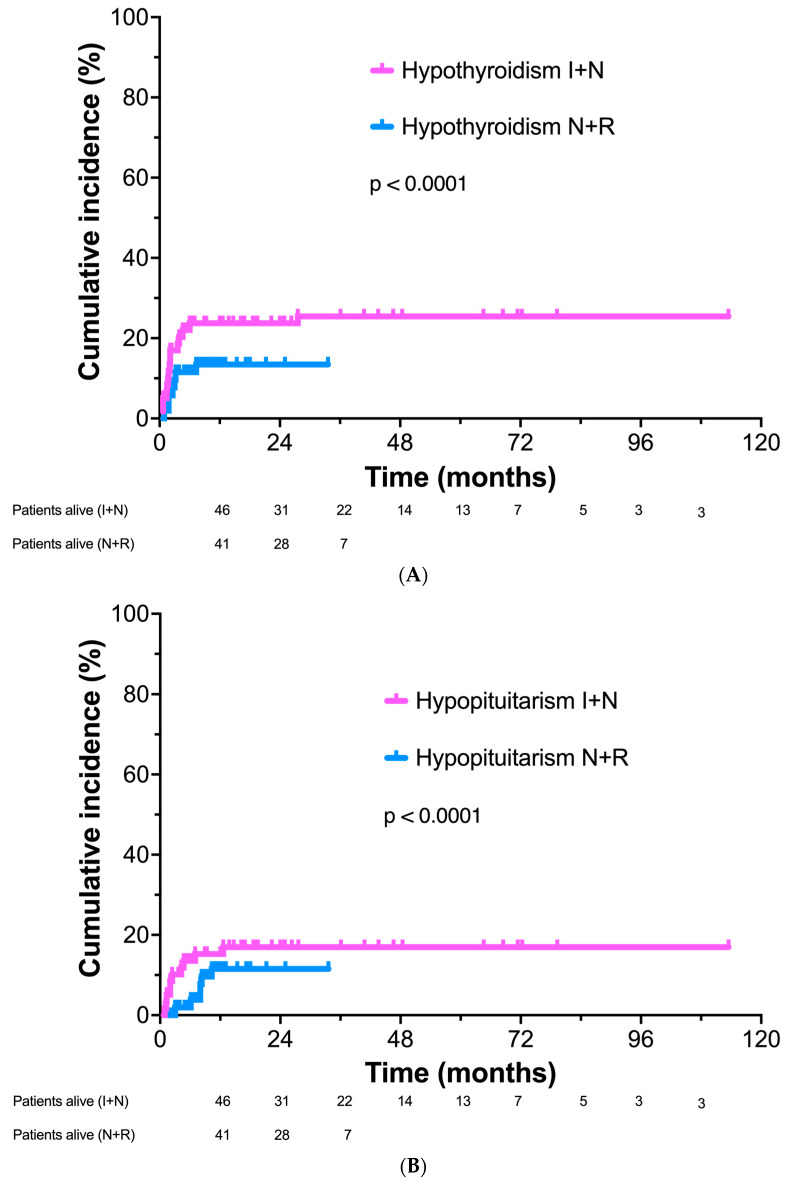
Exploratory analysis of cumulative incidence of endocrinopathy following nivolumab plus relatlimab versus ipilimumab vs. nivolumab. (**A**): hypothyroidism; (**B**): hypopituitarism. The number of patients available for assessment at various times is indicated in the table below the figure. It should be noted that relatlimab-plus-nivolumab therapy had more recent regulatory approval, thus resulting in shorter follow-up duration.

An exploratory analysis was performed to compare the risk of endocrinopathy in our N+R-treated patients (n = 52) with that of a separate cohort of sequential patients treated with I+N for metastatic melanoma (n = 58). Overall, 39.7% of I+N treated patients developed endocrinopathy compared to 25% of patients treated with N+R. The cumulative incidence of hypothyroidism was 25.4 vs. 13.5%, respectively (*p* < 0.0001 by log-rank test) (Figure 3A). The cumulative incidence of hypopituitarism was 17.0 vs. 11.5%, respectively (*p* < 0.0001) (Figure 3B).

### 3.5. Complications of Endocrinopathy

If endocrinopathy was detected on serial monitoring, replacement therapy was instituted. Endocrine replacement therapy was titrated based on the results of endocrine testing. No patients in our series required hospitalization due to the development of endocrinopathy. One patient was hospitalized due to the development of concurrent immunotherapy-related colitis; however, this resolved with appropriate treatment. There was no treatment-related mortality. Following the development of endocrinopathy, no patients recovered endocrine function after completion of planned therapy.

## 4. Discussion

I+N represents a well-established standard of care for the treatment of metastatic melanoma [8]. With a minimum follow-up of 10 years, median overall survival with this treatment was 71.9 months, with a 10-year progression-free survival rate of 31% [8]. These outcomes proved to be superior to those achieved with either CTLA-4 and PD-1 monotherapy. Unfortunately, the side effects of this combination regimen can cause significant autoimmune reactions in virtually any organ system [21]. Thus, this combination produces a variety of serious irAEs, such as rash, pruritus, pneumonitis, hepatitis, colitis, and endocrinopathy in approximately 55% of treated patients [22].

More recently, combination immunotherapy with N+R, targeting PD-1 and LAG-3 respectively, has also demonstrated improved progression-free and overall survival outcomes in patients with metastatic melanoma compared with single-agent nivolumab [9]. Although the clinical effectiveness of N+R therapy has not yet been directly compared to standard I+N therapy in a prospective trial, an ad hoc comparison by Long et al. reported that the overall clinical efficacy of both treatments appeared to be comparable, based on similar PFS and OS [10]. This analysis showed that N+R administration was associated with fewer grade 3–4 treatment-related irAEs than I+N (23% v 61%). N+R was also associated with a lower treatment-discontinuation rate (17% v 41%), suggesting a more favorable safety profile.

Current guidelines vary substantially with regard to routine monitoring of pituitary function. Current National Comprehensive Cancer Network (NCCN) and European Society for Medical Oncology (ESMO) guidelines only suggest pituitary function testing if patients become symptomatic [23,24]. In contrast, the Society for Immunotherapy of Cancer guidelines (SITC) suggest routine thyroid and pituitary function monitoring similar to our institutional practice [25].

We have previously evaluated the risk of endocrinopathy during I+N treatment using screening laboratory tests [26]. This previous study demonstrated an overall endocrinopathy risk of 41.3% with this regimen. This included 22.4% of patients with primary hypothyroidism and 18.9% with hypopituitarism (characterized by central hypothyroidism and secondary hypoadrenalism). Given the potential lower overall incidence of irAE with N+R therapy, the goal of the current study was to evaluate the incidence and timing of endocrinopathy in sequentially screened patients during N+R treatment.

In 52 sequential metastatic melanoma patients treated with first-line N+R therapy, 13 patients developed endocrinopathy on sequential monitoring (25%). Primary hypothyroidism was observed in 13.5%. In addition, 11.5% developed hypopituitarism, resulting in central hypothyroidism and secondary adrenal insufficiency. The median time to onset of hypopituitarism tended to be more delayed than the onset of hypothyroidism (243.5 vs. 79.0 days, respectively). Evaluation of the cumulative onset of endocrinopathy demonstrated distinct temporal patterns between these toxicities with primary hypothyroidism occurring earlier than hypopituitarism following N+R treatment. These findings suggest that thyroid dysfunction may represent an acute manifestation of immune-related endocrine toxicity, whereas pituitary dysfunction generally emerged after more sustained N+R exposure, highlighting the importance of continued longitudinal endocrine surveillance throughout N+R therapy. As reported by other investigators, patients who developed treatment-induced endocrinopathy tended to have improved clinical outcomes (PFS and OS) compared with patients without endocrinopathy [27,28]. However, this association should be interpreted with caution, as patients who develop endocrine irAEs must remain alive and on therapy long enough for these toxicities to occur, introducing the potential for immortal time bias. Therefore, the observed relationship between endocrinopathy and improved clinical outcomes should be considered associative rather than causative. Future studies incorporating landmark analyses are needed to more accurately evaluate the relationship between endocrine toxicity development and survival outcomes. Upon elective discontinuation of treatment in a confirmed remission, none of our patients recovered endocrine function.

There is yet little published real-world data related to the endocrine toxicity of N+R treatment. Kielkowski et al. reported only a 12.5% incidence of hypothyroidism and a 0% incidence of hypopituitarism (versus 14% and 7% with I+N) [29]. Since this was a review of real-world data, it is not clear whether endocrine screening testing was performed in these patients. Our results show a similar rate of hypothyroidism with N+R, compared to other LAG-3 plus PD-1 inhibitor antibody combinations in published clinical trials. In a prior phase III trial of N+R, a 16.6% incidence of hypothyroidism and a 5.9% incidence of hypoadrenalism were reported [9]. A phase I study of fianlimab (anti-LAG-3) plus cemiplimab (anti-PD-1) resulted in a 13.3% incidence of hypothyroidism and a 12.2% incidence of “hypoadrenalism” [30]. In contrast, favezelimab (anti-LAG-3) plus pembrolizumab (anti-PD-1) was reported to result in only a 5% risk of hypothyroidism, but hypopituitarism was not assessed [31]. Differences in reported endocrine toxicity rates across studies may reflect variations in study populations, follow-up duration, endocrine evaluation protocols, and definitions of specific endocrinopathies, particularly among retrospective and real-world analyses. Prospective clinical trials, including RELATIVITY-047, virtually always have prescribed standardized adverse-event monitoring.

It should be noted that there has been variability in clinical trial design in relation to monitoring of pituitary function. In addition, there has been a significant issue related to nomenclature of pituitary abnormalities identified in various study publications (including the functional term “hypopituitarism”, and the anatomic or mechanistic description “hypophysitis”). Many additional authors have described “hypoadrenalism”, although this is almost always secondary hypoadrenalism due to the lack of pituitary ACTH secretion. It should be noted that primary hypoadrenalism following checkpoint inhibitor treatment appears to be rare. Another issue is that the CTCAE toxicity criteria are based on the functional severity of endocrine toxicities. Thus, these criteria are not useful in defining the incidence, timing of onset, and persistence of endocrine abnormalities when detected in a pre-symptomatic state. Since we performed routine endocrine monitoring of all patients, including for development of hypopituitarism, our study is more likely to provide a more accurate assessment of this risk following N+R therapy.

The pathogenesis of checkpoint inhibitor-induced endocrinopathy remains unclear. Regulatory T cells (Tregs) and T cell inhibitory checkpoints should prevent immune reactions against self-antigens. There is developing evidence that genetic susceptibility to autoimmune endocrinopathy may also be a significant contributory factor to the development of autoreactive CD4 and CD8 T cells [32].

T cell activation by ICI also mediates the release of inflammatory cytokines, such as gamma interferon. These cytokines may upregulate MHC class II expression on thyrocytes, as well as other endocrine tissues [33,34]. This potentially allows thyrocytes to present thyroid antigens, such as thyroglobulin (Tg), thyroid peroxidase (TPO), and the TSH receptor (TSHR), directly to CD4^+^ helper T cells. The net result appears to be increased T cell autoreactivity against endocrine tissues, resulting in endocrinopathy.

In an exploratory analysis in cohorts of sequentially treated patients, N+R treatment displayed a lower incidence of hypopituitarism and hypothyroidism than I+N. This difference proved to be statistically significant by log-rank test (*p*-value < 0.0001).

The strengths of the current study include routine laboratory screening for endocrinopathy on each clinic visit for all patients consistently treated with N+R. In addition, all patients were managed in a consistent fashion by a single medical oncologist. Weaknesses of the study include a relatively small sample size and a relatively short duration of follow-up. Moreover, the small sample size and number of endocrine events limited the statistical power of our exploratory analysis versus I+N treatment.

Other hypothalamic and pituitary hormones were not routinely measured, due to cost and insurance reimbursement issues. Screening for adrenal and pituitary function using random cortisol measurements, rather than standardized morning cortisol levels, may have somewhat reduced the sensitivity for detection of hypopituitarism, requiring confirmatory testing. Future studies focusing on diurnal variation in pituitary hormones following N+R therapy will need to be performed. While our study included all sequential patients treated in our clinic with first-line N+R and I+N, this comparison was non-randomized and was retrospective in nature. Therefore, these findings should be interpreted with caution, as the two groups potentially could differ in baseline patient characteristics, treatment era, follow-up duration, disease burden, prior therapies, and other clinical factors that could affect the development of endocrinopathy. Additionally, no adjustment for potential confound variables was performed due to the limited sample size. As a result, comparison between N+R and I+N should be considered hypothesis-generating, and larger prospective studies with appropriate stratification of baseline characteristics are needed to better characterize potential differences in endocrine toxicity risk between these treatment regimens.

A larger sample size is likely to have shown a stronger statistical relationship between endocrine toxicity and OS and PFS. However, survival analyses evaluating the association between endocrinopathy and improved outcomes are limited by the retrospective study design and potential immortal time bias, as patients with longer treatment exposure may have a greater propensity to develop pituitary toxicity. Future prospectives studies using landmark analysis are needed to confirm whether endocrinopathy represents an independent predictor of improved treatment response. It is also unclear from this retrospective analysis whether endocrinopathy would have eventually resolved in some patients. Thus, this study should be considered hypothesis-generating and should be confirmed in prospective trials.

## 5. Conclusions

We evaluated endocrine toxicity as an irAE following first-line nivolumab-plus-relatlimab therapy in patients with metastatic cutaneous melanoma. A total of 25% of N+R treated patients developed biochemical evidence of endocrinopathy. This included 13.5% with hypothyroidism and 11.5% with hypopituitarism. The median onset of hypothyroidism was early (median 79 days), while the onset of hypopituitarism tended to be more delayed, following more prolonged treatment (median 244 days). Early detection of endocrinopathy and intervention prevented hospitalizations and treatment delays. Late onset of endocrinopathy following elective ICI treatment discontinuation upon achieving remission was uncommon (3.9%). An exploratory analysis suggested that there was a significantly lower risk of endocrinopathy following N+R treatment than following I+N administration. Routine monitoring of pituitary, adrenal and thyroid function during combination N+R therapy is strongly recommended to allow early detection of endocrinopathy and intervention while patients remain presymptomatic.

## Data Availability

The de-identified data underlying this article will be shared on reasonable request to the corresponding author.

## Supplementary Materials

The following supporting information can be downloaded at: https://www.mdpi.com/article/10.3390/cancers18142349/s1, Table S1: Patient Demographics Stratified by Endocrinopathy Status; Table S2: Patient Outcomes Stratified by Endocrinopathy Status.

## Abbreviations

The following abbreviations are used in this manuscript:

ACTH	Adrenocorticotropic hormone
BORR	Best observed response
CR	Complete remission
FT4	Free-T4 (tetraiodothyronine)
DOB	Date of birth
HIPAA	Health Insurance Portability and Accountability Act
ICI	Immune checkpoint inhibitors
I+N	Ipilimumab plus nivolumab
irAE	Immune-related adverse events
IRB	Institutional review board
LAG-3	Lymphocyte-activation gene 3
LDH	Lactate dehydrogenase
N+R	Nivolumab plus relatlimab
OS	Overall survival
PFS	Progression-free survival
PD	Progressive disease
PR	Partial response
SD	Stable disease
Tg	Thyroglobulin
TPO	Thyroid peroxidase
TSH	Thyroid stimulating hormone
TSHR	Thyroid stimulating hormone receptor

## References

[1] Siegel R.L., Kratzer T.B., Giaquinto A.N., Sung H., Jemal A. (2025). Cancer statistics, 2025. CA Cancer J. Clin..

[2] Chapman P.B., Einhorn L.H., Meyers M.L., Saxman S., Destro A.N., Panageas K.S., Begg C.B., Agarwala S.S., Schuchter L.M., Ernstoff M.S. (1999). Phase III multicenter randomized trial of the Dartmouth regimen versus dacarbazine in patients with metastatic melanoma. J. Clin. Oncol..

[3] Chiarion-Sileni V., Guida M., Ridolfi L., Romanini A., Del Bianco P., Pigozzo J., Brugnara S., Colucci G., Ridolfi R., De Salvo G.L. (2011). Central nervous system failure in melanoma patients: Results of a randomised, multicentre phase 3 study of temozolomide- and dacarbazine- based regimens. Br. J. Cancer.

[4] Korn E.L., Liu P.Y., Lee S.J., Chapman J.A., Niedzwiecki D., Suman V.J., Moon J., Sondak V.K., Atkins M.B., Eisenhauer E.A. (2008). Meta-analysis of phase II cooperative group trials in metastatic stage IV melanoma to determine progression-free and overall survival benchmarks for future phase II trials. J. Clin. Oncol..

[5] Sandru A., Voinea S., Panaitescu E., Blidaru A. (2014). Survival rates of patients with metastatic malignant melanoma. J. Med. Life.

[6] Allison J.P. (2015). Immune Checkpoint Blockade in Cancer Therapy: The 2015 Lasker-DeBakey Clinical Medical Research Award. JAMA.

[7] Rothschild S.I., Thommen D.S., Moersig W., Muller P., Zippelius A. (2015). Cancer immunology-development of novel anti-cancer therapies. Swiss Med. Wkly..

[8] Wolchok J.D., Chiarion-Sileni V., Rutkowski P., Cowey C.L., Schadendorf D., Wagstaff J., Queirolo P., Dummer R., Butler M.O., Hill A.G. (2025). Final, 10-Year Outcomes with Nivolumab plus Ipilimumab in Advanced Melanoma. N. Engl. J. Med..

[9] Tawbi H.A., Hodi F.S., Lipson E.J., Schadendorf D., Ascierto P.A., Matamala L., Castillo Gutierrez E., Rutkowski P., Gogas H., Lao C.D. (2025). Three-Year Overall Survival With Nivolumab Plus Relatlimab in Advanced Melanoma From RELATIVITY-047. J. Clin. Oncol..

[10] Long G.V., Lipson E.J., Hodi F.S., Ascierto P.A., Larkin J., Lao C., Grob J.J., Ejzykowicz F., Moshyk A., Garcia-Horton V. (2024). First-Line Nivolumab Plus Relatlimab Versus Nivolumab Plus Ipilimumab in Advanced Melanoma: An Indirect Treatment Comparison Using RELATIVITY-047 and CheckMate 067 Trial Data. J. Clin. Oncol..

[11] Yoo W.S., Ku E.J., Lee E.K., Ahn H.Y. (2023). Incidence of Endocrine-Related Dysfunction in Patients Treated with New Immune Checkpoint Inhibitors: A Meta-Analysis and Comprehensive Review. Endocrinol. Metab..

[12] Almutairi A.R., McBride A., Slack M., Erstad B.L., Abraham I. (2020). Potential Immune-Related Adverse Events Associated With Monotherapy and Combination Therapy of Ipilimumab, Nivolumab, and Pembrolizumab for Advanced Melanoma: A Systematic Review and Meta-Analysis. Front. Oncol..

[13] Johnson J., Goldner W., Abdallah D., Qiu F., Ganti A.K., Kotwal A. (2023). Hypophysitis and Secondary Adrenal Insufficiency From Immune Checkpoint Inhibitors: Diagnostic Challenges and Link With Survival. J. Natl. Compr. Cancer Netw..

[14] Perez L., Samlowski W., Lopez-Flores R. (2022). Outcome of Elective Checkpoint Inhibitor Discontinuation in Patients with Metastatic Melanoma Who Achieved a Complete Remission: Real-World Data. Biomedicines.

[15] Panning A., Samlowski W., Allred G. (2023). Lack of Influence of Non-Overlapping Mutations in BRAF, NRAS, or NF1 on 12-Month Best Objective Response and Long-Term Survival after Checkpoint Inhibitor-Based Treatment for Metastatic Melanoma. Cancers.

[16] Eisenhauer E.A., Therasse P., Bogaerts J., Schwartz L.H., Sargent D., Ford R., Dancey J., Arbuck S., Gwyther S., Mooney M. (2009). New response evaluation criteria in solid tumours: Revised RECIST guideline (version 1.1). Eur. J. Cancer.

[17] Kaplan E.L., Meier P. (1958). Nonparametric Estimation from Incomplete Observations. J. Am. Stat. Assoc..

[18] Gehan E.A. (1965). A Generalized Wilcoxon Test for Comparing Arbitrarily Singly-Censored Samples. Biometrika.

[19] Wilcoxon F. (1946). Individual comparisons of grouped data by ranking methods. J. Econ. Entomol..

[20] Mann H.B., Whitney D.R. (1947). On a Test of Whether One of Two Random Variables Is Stochastically Larger Than the Other. Ann. Math. Stat..

[21] Martins F., Sofiya L., Sykiotis G.P., Lamine F., Maillard M., Fraga M., Shabafrouz K., Ribi C., Cairoli A., Guex-Crosier Y. (2019). Adverse effects of immune-checkpoint inhibitors: Epidemiology, management and surveillance. Nat. Rev. Clin. Oncol..

[22] Larkin J., Chiarion-Sileni V., Gonzalez R., Grob J.J., Cowey C.L., Lao C.D., Schadendorf D., Dummer R., Smylie M., Rutkowski P. (2015). Combined Nivolumab and Ipilimumab or Monotherapy in Untreated Melanoma. N. Engl. J. Med..

[23] Thompson J.A., Schneider B.J., Brahmer J., Andrews S., Armand P., Bhatia S., Budde L.E., Costa L., Davies M., Dunnington D. (2020). NCCN Guidelines Insights: Management of Immunotherapy-Related Toxicities, Version 1.2020. J. Natl. Compr. Cancer Netw..

[24] Haanen J., Obeid M., Spain L., Carbonnel F., Wang Y., Robert C., Lyon A.R., Wick W., Kostine M., Peters S. (2022). Management of toxicities from immunotherapy: ESMO Clinical Practice Guideline for diagnosis, treatment and follow-up. Ann. Oncol..

[25] Puzanov I., Diab A., Abdallah K., Bingham C.O., Brogdon C., Dadu R., Hamad L., Kim S., Lacouture M.E., LeBoeuf N.R. (2017). Managing toxicities associated with immune checkpoint inhibitors: Consensus recommendations from the Society for Immunotherapy of Cancer (SITC) Toxicity Management Working Group. J. Immunother. Cancer.

[26] Hasan M., Samlowski W., Nakhle S. (2026). Systematic monitoring identified a high incidence of hypopituitarism following combined ipilimumab plus nivolumab therapy for metastatic melanoma. Front. Endocrinol..

[27] Garcia-Goni M., Vazquez Gutierrez B., Sanmamed M.F., Martin-Algarra S., Luis Perez-Gracia J., Olmedo M., Chumbiauca E., Martin-Calvo N., Galofre J.C. (2024). Thyroid dysfunction caused by immune checkpoint inhibitors improves cancer outcomes. Endocr. Relat. Cancer.

[28] Takagi T., Yoshida K., Kondo T., Fukuda H., Ishihara H., Kobayashi H., Iizuka J., Ishida H., Tanabe K. (2021). Hypopituitarism in patients with metastatic renal cell carcinoma treated with ipilimumab and nivolumab combination therapy. Jpn. J. Clin. Oncol..

[29] Kielkowski B., Mansour D., Ebbert B., Seago K., Wen S., Li H., Barrett C. (2026). Real world comparison of immune-related adverse events with nivolumab-relatlimab versus ipilimumab-nivolumab in patients with advanced cutaneous melanoma. J. Oncol. Pharm. Pract..

[30] Hamid O., Lewis K.D., Weise A., McKean M., Papadopoulos K.P., Crown J., Kim T.M., Lee D.H., Thomas S.S., Mehnert J. (2024). Phase I Study of Fianlimab, a Human Lymphocyte Activation Gene-3 (LAG-3) Monoclonal Antibody, in Combination With Cemiplimab in Advanced Melanoma. J. Clin. Oncol..

[31] Garralda E., Sukari A., Lakhani N.J., Patnaik A., Lou Y., Im S.A., Golan T., Geva R., Wermke M., de Miguel M. (2022). A first-in-human study of the anti-LAG-3 antibody favezelimab plus pembrolizumab in previously treated, advanced microsatellite stable colorectal cancer. ESMO Open.

[32] Gandarillas S., Newland E.S., Toppmeyer D., Stephenson R., Denzin L., Dasgeb B. (2023). HLA inherence as a potential parameter in checkpoint inhibitor-associated autoimmune adverse event assessment. Front. Med..

[33] Holling T.M., Schooten E., van Den Elsen P.J. (2004). Function and regulation of MHC class II molecules in T-lymphocytes: Of mice and men. Hum. Immunol..

[34] Taube J.M., Young G.D., McMiller T.L., Chen S., Salas J.T., Pritchard T.S., Xu H., Meeker A.K., Fan J., Cheadle C. (2015). Differential Expression of Immune-Regulatory Genes Associated with PD-L1 Display in Melanoma: Implications for PD-1 Pathway Blockade. Clin. Cancer Res..

