# Supplementary material for: Incidence and Clinical Impact of Endocrinopathy Following First-Line Nivolumab-Plus-Relatlimab Therapy for Metastatic Melanoma
Source: Cancers (Basel). 2026 Jul 21;18(14):2349. doi: 10.3390/cancers18142349 (PMC13406166; doi:10.3390/cancers18142349)
Supplement: Supplementary file 1 [file cancers-18-02349-s001.zip › Table S1.pdf]

Supplemental Data: Table S1 Patient Demographics Stratified by Endocrinopathy Status

Patients With Endocrinopathy (n=13)

UPN	Age at initiation	Gender (M/F)	Race	Potential driver mutations	LDH (CKI start)	Sites of Metastases
6	76.2	M	W		162	
13	55.5	F	W	BRAF V600R	183	Skin, Breast, Lung
18	75.9	M	W	BRAF V600K	236	
28	78.4	M	W		175	
29	42.2	F	W		160	
30	78.1	F	W			
36	85.1	F	W		231	Skin
39	91.2	F	O	BRAF V600E	160	Skin
40	65.2	M	W		168	Skin
42	64.1	M	W		172	Skin
46	49.7	M	O		155	Skin
49	50.2	M	D		155	Skin
50	44.8	M	O		257	Skin

Patients Without Endocrinopathy (n=39)

UPN	Age at initiation	Gender (M/F)	Race	Potential driver mutations	LDH (CKI start)	Sites of Metastases
1	84.8	M	H	BRAF V600E	151	lung, skin, abdominal, adrenal
2	82.7	F	W	TP53 E285K	237	Lung
3	85.9	M	W	BRAF Mutation: Wild type	158	

4	39.3	F	W	BRAF Mutation: Wild type	193	
5	77.7	F	W		193	
7	73.9	M	W	NF1 Mutation	154	pulmonary, liver, skin
8	71.3	F	W		251	lung, liver, cervical/supraclavicular/mediastinal/retroperitoneal LN
9	41.8	M	W		252	
10	78.4	M	W	BRAF Mutation: Wild type	334	
11	51.6	F	W		163	
12	80.6	F	D		168	Brain
14	67.1	M	D		115	
15	64.5	F	W	BRAF V600E	209	Brain
16	64.2	M	W	NRAS, NF1 mutations	170	Bones of skull
17	87.2	F	W		225	
19	86.1	M	W	BRAF V600R	190	Lung, skin
20	77.7	M	D	NRAS	571	Prostate, Skin, Spleen, Lung, Liver
21	80.0	M	W	NRAS	204	
22	74.2	M	W		241	
23	71.9	M	W	BRAF G469E	195	
24	83.9	M	W		267	
25	72.2	F	W		265	
26	57.3	M	W		199	
27	47.4	M	W	BRAF V600E	200	
31	92.6	M	W	BRAF V600K	183	
32	60.3	F	A		242	pleural lesions, mediastinal nodes, pelvic LN, bone metastases

33	74.7	F	D	KIT D820A mutation	185	Skin
34	87.6	M	D		213	liver, skin, possible lung
35	43.4	M	W		111	oral, lung, skin
37	64.0	M	D		188	
38	68.2	F	W	BRAF V471F (LOF)	243	brain, skin
41	76.4	M	W	BRAF deletion exon 2/3, p10 loss	212	skin
43	64.1	M	W	NRAS Q61R	153	Skin
44	78.5	M	W	CKIT L576P	170	skin, brain
45	71.3	M	W	BRAF V600E	171	Skin
47	73.5	M	W	BRCA2 N2208fs	340	Skin
48	70.1	M	W	BRAF V600K	150	Skin
51	81.1	M	W	NF1 K144Q	217	Skin
52	61.6	M	W	NRAS Q16R	223	Skin
