# Supplementary material for: Incidence and Clinical Impact of Endocrinopathy Following First-Line Nivolumab-Plus-Relatlimab Therapy for Metastatic Melanoma
Source: Cancers (Basel). 2026 Jul 21;18(14):2349. doi: 10.3390/cancers18142349 (PMC13406166; doi:10.3390/cancers18142349)
Supplement: Supplementary file 1 [file cancers-18-02349-s001.zip › Table S2.pdf]

Supplemental Data Table S2: Patient Outcomes Stratified by Endocrinopathy Status

Patients With Endocrinopathy (n=13)

UPN	Age	Gender (M/F)	Race / Ethnicity	Baseline LDH	Pre-existing hypothyroidism	New Hypothyroidism (T) or Hypopituitarism (P)	Onset (d)	PFS (mo)	OS (mo)	Current Status	Endocrinopathy
6	76.2	M	C	162		P	187	27.2	27.2	NED	yes
13	55.5	F	C	183		T	91	4.5	7.0	AWD	yes
18	75.9	M	C	236		T	27	2.5	3.5	AWD	yes
28	78.4	M	C	175		T	56	28.1	28.1	NED	yes
29	42.2	F	C	160		P	92	3.1	3.1	AWD	yes
30	78.1	F	C			P	252	10.9	10.9	NED	yes
36	85.1	F	C	231		T	55	9.9	9.9	AWD	yes
39	91.2	F	C	160		T	99	15.2	18.3	AWD	yes
40	65.2	M	C	168		P	315	6.0	10.5	AWD	yes
42	64.1	M	C	172		P	243	14.4	14.4	NED	yes
46	49.7	M	C	155		P	244	13.3	13.3	NED	yes
49	50.2	M	C	155		T	224	11.4	11.4	AWD	yes
50	44.8	M	C	257			189	11.9	11.9	NED	yes

Patients Without Endocrinopathy (n=39)

UPN	Age	Gender (M/F)	Race / Ethnicity	Baseline LDH	Pre-existing hypothyroidism	New Hypothyroidism (T) or Hypopituitarism (P)	Onset (d)	PFS (mo)	OS (mo)	Current Status	Endocrinopathy
1	84.8	M	H	151	Y			3.2	9.1	AWD	
2	82.7	F	C	237	Y			25.4	25.4	NED	
3	85.9	M	C	158	Y			3.0	3.0	AWD	
4	39.3	F	C	193				15.6	15.6	NED	
5	77.7	F	C	193				12.5	12.5	NED	
7	73.9	M	C	154				12.8	12.8	DWD	
8	71.3	F	C	251				10.8	10.8	NED	
9	41.8	M	C	252				11.4	11.4	NED	
10	78.4	M	C	334				1.8	1.8	AWD	
11	51.6	F	C	163	Y			4.2	21.6	NED	
12	80.6	F	C	168	Y			5.6	12.7	AWD	
14	67.1	M	C	115				0.9	0.9	AWD	
15	64.5	F	C	209				8.6	17.5	DWD	
16	64.2	M	C	170				9.8	11.4	AWD	
17	87.2	F	C	225				2.1	3.1	DWD	
19	86.1	M	C	190				1.9	1.9	AWD	
20	77.7	M	C	571				0.7	0.7	DWD	
21	80.0	M	C	204				3.5	6.3	DWD	
22	74.2	M	C	241	Y			4.9	4.9	AWD	
23	71.9	M	C	195				2.0	2.0	DWD	
24	83.9	M	C	267	Y			18.3	18.3	NED	
25	72.2	F	C	265	Y			34.2	34.2	NED	

26	57.3	M	C	199				1.6	1.6	AWD	
27	47.4	M	C	200				7.0	7.0	AWD	
31	92.6	M	C	183				10.7	9.9	AWD	
32	60.3	F	A	242				3.8	3.8	AWD	
33	74.7	F	H	185		T	80	2.6	8.7	AWD	
34	87.6	M	C	213				5.8	5.8	DWD	
35	43.4	M	C	111	Y			3.0	5.6	AWD	
37	64.0	M	C	188				1.9	1.9	AWD	
38	68.2	F	C	243				2.5	2.5	DWD	
41	76.4	M	C	212				3.7	6.8	DWD	
43	64.1	M	C	153				4.0	4.0	NED	
44	78.5	M	C	170				13.4	13.4	NED	
45	71.3	M	C	171				7.5	7.5	AWD	
47	73.5	M	C	340				13.3	13.3	NED	
48	70.1	M	C	150				2.8	3.7	AWD	
51	81.1	M	C	217				9.6	9.6	NED	
52	61.6	M	C	223				8.2	8.2	AWD	

UPN, Unique Patient Number; M, Male; F, Female; C, Caucasian; H, Hispanic; A, Asian; NED, No Evidence of Disease; AWD, Alive With Disease; DWD, Died With Disease; DOD, Died of Disease; Y, Yes; T, Hypothyroidism; P, Hypopituitarism.
